# Melatonin Improves the Photosynthetic Carbon Assimilation and Antioxidant Capacity in Wheat Exposed to Nano-ZnO Stress

**DOI:** 10.3390/molecules22101727

**Published:** 2017-10-18

**Authors:** Zhiyu Zuo, Luying Sun, Tianyu Wang, Peng Miao, Xiancan Zhu, Shengqun Liu, Fengbin Song, Hanping Mao, Xiangnan Li

**Affiliations:** 1 Key Laboratory of Modern Agricultural Equipment and Technology, Ministry of Education/High-tech Key Laboratory of Agricultural Equipment and Intelligence of Jiangsu Province, School of Agricultural Equipment and Engineering, Jiangsu University, Zhenjiang 212013, China; zuozy@ujs.edu.cn (Z.Z.); wangtianyu1014@126.com (T.W.); miaopengly@163.com (P.M.); maohp@ujs.edu.cn (H.M.); 2 Northeast Institute of Geography and Agroecology, Chinese Academy of Sciences, Changchun 130102, China; sunluy1@126.com (L.S.); zhuxiancan@iga.ac.cn (X.Z.); lsq@iga.ac.cn (S.L.); songfb@iga.ac.cn (F.S.)

**Keywords:** chlorophyll fluorescence, phytotoxicity, *Triticum aestivum*, antioxidant, nanoparticle

## Abstract

The release of nanoparticles into the environment is inevitable, which has raised global environmental concern. Melatonin is involved in various stress responses in plants. The present study investigated the effects of melatonin on photosynthetic carbon (C) assimilation and plant growth in nano-ZnO stressed plants. It was found that melatonin improved the photosynthetic C assimilation in nano-ZnO stressed wheat plants, mainly due to the enhanced photosynthetic energy transport efficiency, higher chlorophyll concentration and higher activities of Rubisco and ATPases. In addition, melatonin enhanced the activities of antioxidant enzymes to protect the photosynthetic electron transport system in wheat leaves against the oxidative burst caused by nano-ZnO stress. These results suggest that melatonin could improve the tolerance of wheat plants to nano-ZnO stress.

## 1. Introduction

Nanoparticles (NPs) are also known as particulate nanomaterials, which have at least one dimension in the nanoscale (1–100 nm) [1]. A dramatic increase in the application of NPs in nanomedicine and nanopharmacology increased the likelihood of NPs release into the environment, which mostly contain heavy metals [2,3]. The soil and water contamination with NPs has significant impacts on animal and plant systems [4]. The NPs lead to physical and chemical damage to plants, not only by attaching to the plant surface, but also by entering the plants through the lateral root junctions [5]. Especially, the NPs, which has a smaller size than the cell wall pore, can cross the cell wall and reach the plasma membrane, hence affecting the cell metabolic pathways [6].

It has been documented that some kinds of NPs can be easily uptaken [7], transported [3,8] and accumulated [4,9] in plant organs, and alter the plant physiological and biochemical processes [1,3,10], hence influence plant growth and development [1,11]. The chemical interactions between NPs and plants involve the production of reactive oxygen species (ROS) [2,4], lipid peroxidation [12], and changes of protein activity [4]. In plant organs, NPs act as metal ions and react with sulfhydryl and carboxyl groups of proteins, and thus change the activities of enzymes [2]. In addition, NPs can trigger a severe oxidative burst, due to the overproduction of ROS [13]. This results in oxidative damage to nucleic acid and protein, which affect the efficiency of photosynthetic apparatus and carbon (C) assimilation process [14].

ZnO NPs are among the most popular NPs in various applications, such as medicines, pigments, and batteries [15]. The toxicity effects of ZnO NPs have been reported in several plant species, including wheat (*Triticum aestivum* L.) [16] and buckweed (*Spirodela punctuta*) [17]. For instance, the exposure to ZnO NPs caused the increased generation of ROS and reactive nitrogen species in duckweed [18]. In rice, ZnO NPs (10–1000 mg L^−1^) significantly reduced the root length at early growing stages [19]. In *Arabidopsis*, it was found that 200 and 300 mg L^−1^ ZnO NPs treatments decreased the plant growth by about 20 and 80%, respectively, in relation to the control [1]. In addition, it was demonstrated that the toxicity effects of ZnO NPs observed in *Arabidopsis* was due to the inhibition of the expression of chlorophyll synthesis genes and photosystem structure genes, leading to the inhibition of chlorophylls biosynthesis and the decrease in photosynthesis efficiency [1].

Melatonin (*N*-acetyl-5-methoxytryptamine) has been demonstrated to improve the tolerance to biotic and abiotic stress in different plant species [20,21,22]. Exogenous application of melatonin can stimulate plant growth [20], enhance photosynthetic C assimilation [23], and promote cellular protein protection [24]. Also, melatonin functions as an antioxidant to enhance the antioxidant capacity of organelles, when the plant exposed to abiotic stress [25,26,27,28]. Melatonin and its metabolites regulate a large number of antioxidative and pro-oxidative enzymes, resulting in a reduction in oxidative damage [29]. Moreover, melatonin has been reported to modulate the stress responsive genes, such as C-repeat-binding factors (*CBFs*), to improve resistance to both abiotic and biotic stress [30]. It was documented that melatonin confers plant tolerance to heavy metal stress, such as cadmium (Cd) and copper [31,32,33]. For example, melatonin modulates heavy metal transporters to decrease the Cd accumulation in plants [32,33]. In addition, melatonin is involved in selenium-induced Cd tolerance via the regulation of Cd detoxification [34]. However, the information regarding the effects of melatonin on the responses of plant to NPs is limited.

The objectives of this study were to investigate the interactive effects of melatonin and nano-ZnO on plant growth, photosynthetic C assimilation, and antioxidant capacity in wheat plants. It was hypothesized that (i) exogenous melatonin application would improve wheat plant growth under nano-ZnO stress and (ii) melatonin would enhance the antioxidant capacity and improve photosynthetic C assimilation in the nano-ZnO stressed wheat plants.

## 2. Results

### 2.1. Effect of Melatonin on Wheat Plant Growth under Nano-ZnO Stress

**Figure 1:**
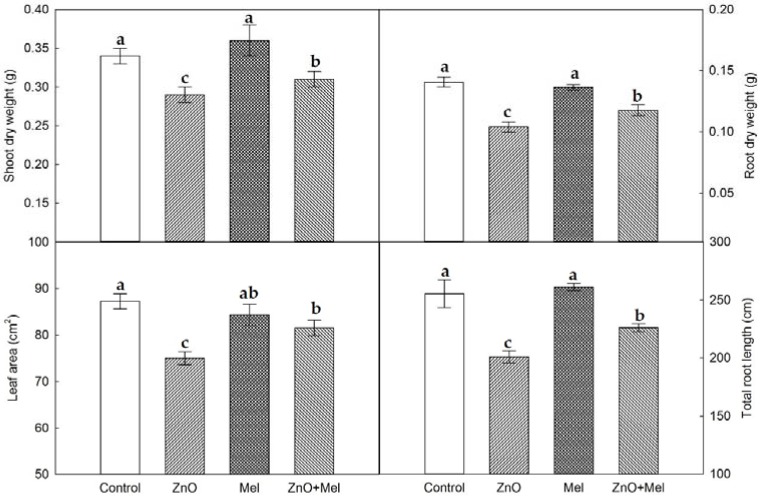
Plant growth parameters in wheat as affected by melatonin application and Zinc Oxide (ZnO) nanoparticles. Mel, melatonin. Data represent the mean ± SE of three replicates. Different letters indicate significant differences (*p* < 0.05).

After 45 days of growth, ZnO NPs stressed plants had significant lower shoot dry weight (by 12.9%) and root dry weight (by 26.3%), decreased leaf area (by 13.7%) and total root length (by 10.7%), compared with the control plants (Figure 1), suggesting that ZnO NPs (250 mg L^−1^) inhibited wheat plant growth. However, under nano-ZnO stress conditions, melatonin treated plants showed significantly higher dry weight and total root length, and larger leaf area, compared with the plants without melatonin treatment (ZnO).

### 2.2. Effect of Melatonin on Chlorophyll Concentration, Gas Exchange, and Chlorophyll a Fluorescence under Nano-ZnO Stress

**Figure 2:**
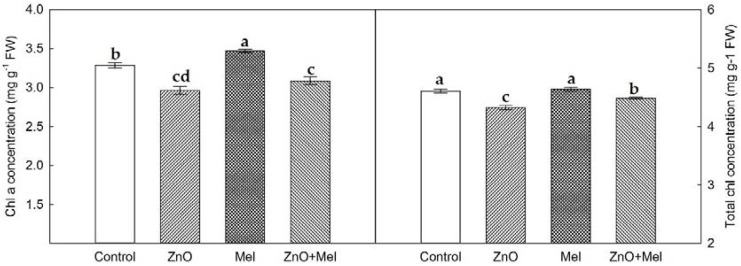
Chlorophyll concentrations in the last fully expanded leaf in wheat as affected by melatonin application and Zinc Oxide (ZnO) nanoparticles. Mel, melatonin. Data represent the mean ± SE of three replicates. Different letters indicate significant differences (*p* < 0.05).

The ZnO NPs reduced the Chl and total Chl concentrations in wheat plants, in relation to the control (Figure 2). Melatonin application significantly increased the total Chl concentration in the last fully expanded leaves exposed to the nano-ZnO stress. In addition, the melatonin treated plants possessed higher Chl a concentration compared with the non-melatonin treated plants, though the difference was not statistically significant.

**Figure 3:**
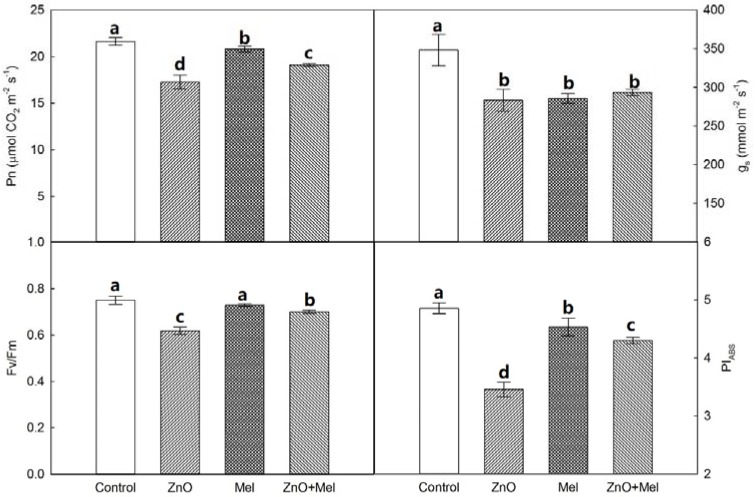
Net photosynthetic rate (Pn), stomatal conductance (*g_s_*), maximum quantum efficiency of photosystem II (Fv/Fm), and performance index (PI_ABS_) in the last fully expanded leaf in wheat as affected by melatonin application and Zinc Oxide (ZnO) nanoparticles. Mel, melatonin. Data represent the mean ± SE of three replicates. Different letters indicate significant differences (*p* < 0.05).

The net photosynthetic rate (Pn) and stomatal conductance (*g_s_*) of the last fully expanded leaves in wheat were both depressed by the nano-ZnO stress (Figure 3). Melatonin application improved significantly Pn of the wheat plants exposed to nano-ZnO stress. However, the *g_s_* of wheat plants was not affected by the melatonin treatment under nano-ZnO stress. A similar trend as Pn was found in the chlorophyll a fluorescence parameters of wheat plants under nano-ZnO stress. For instance, the maximum quantum efficiency of photosystem II (Fv/Fm) and the performance index (PI_ABS_) were reduced significantly by the nano-ZnO treatment, while increased by the melatonin treatment.

**Figure 4:**
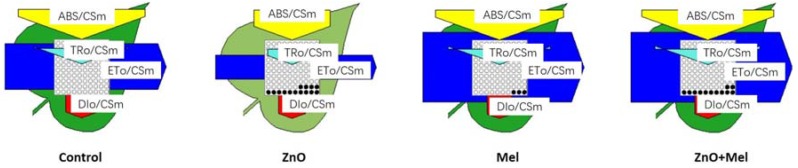
Energy pipeline leaf model of phenomenological fluxes (per cross-section, CS) in the last fully expanded leaf in wheat as affected by melatonin application and Zinc Oxide (ZnO) nanoparticles. Mel, melatonin. Data represent the mean ± SE of three replicates. Each relative value is drawn by the width of the corresponding arrow, standing for a parameter. Empty and full black circles indicate, respectively, the percentage of active (QA reducing) and non-active (non-QA reducing) reaction centres of PS II. TRo/CSm, trapped energy flux per CS; ETo/CSm, electron transport flux per CS; ABS/CSm, absorption flux per CS; DIo/CSm, non-photochemical quenching per CS.

In this study, the leaf model of phenomenological energy fluxes per cross-section was used to visualize the derived parameters (Figure 4). The electron transport in PS II cross-section (ETo/CSm), the absorption flux per cross-section (ABS/CSm) and the trapped energy flux per PS II cross-section (TRo/CSm) were all decreased remarkably by the nano-ZnO stress. In addition, the density of active reaction centres (RCs), as indicated by the number of open circles, was also reduced by the nano-ZnO stress. Melatonin treatment slightly decreased the inactive RC density, and it significantly enhanced the TRo/CSm and ETo/CSm in wheat plants exposed to the nano-ZnO stress.

### 2.3. Responses of Rubisco Activities to Melatonin Application and Nano-ZnO Stress

**Figure 5:**
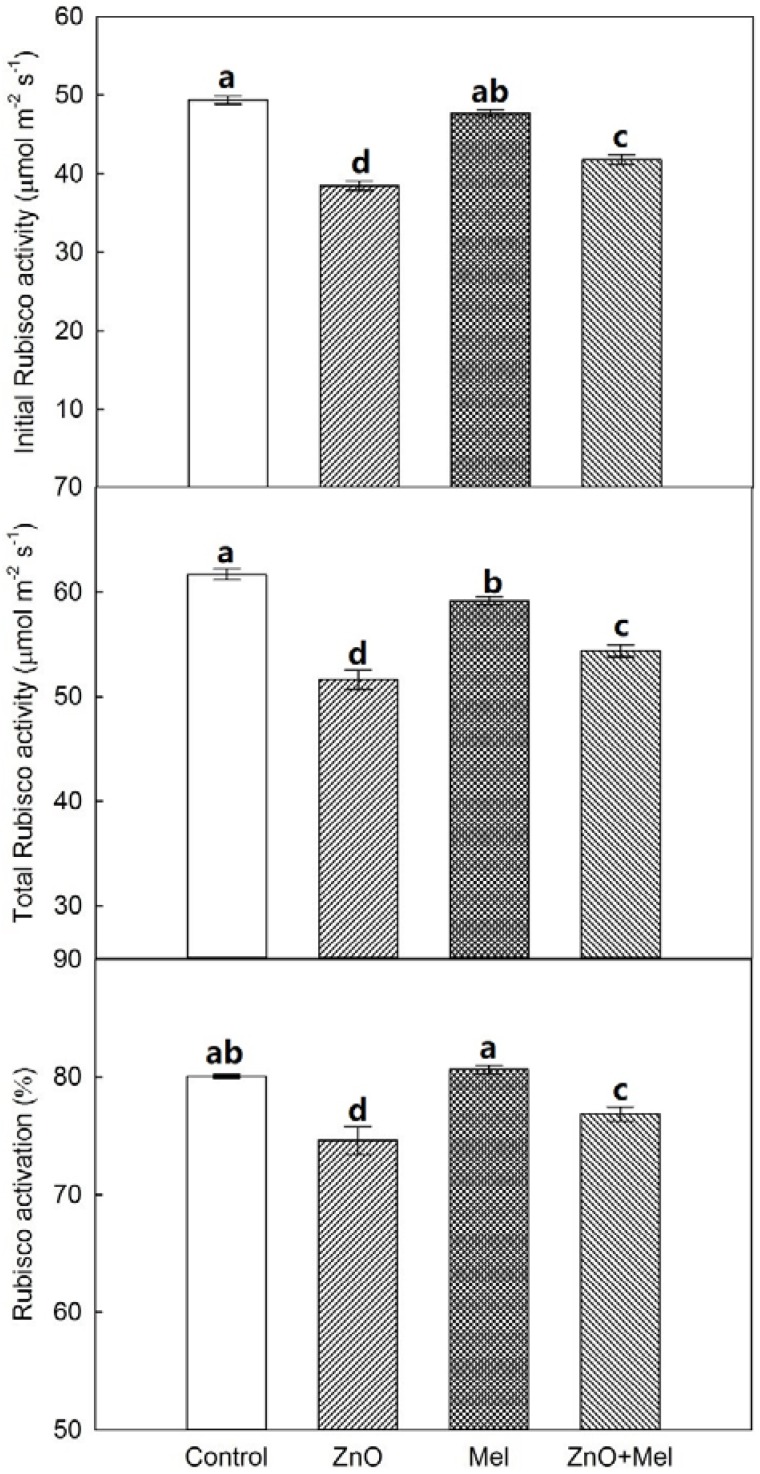
Initial and total Rubisco activities and activation in the last fully expanded leaf in wheat as affected by melatonin application and Zinc Oxide (ZnO) nanoparticles. Mel, melatonin. Data represent the mean ± SE of three replicates. Different letters indicate significant differences (*p* < 0.05).

The initial and total Rubisco (ribulose-1,5-bisphosphate carboxylase/oxygenase) activities were depressed significantly in ZnO treatment, compared with the control (Figure 5). Melatonin treatment significantly increased the activities of initial and total Rubisco in wheat exposed to nano-ZnO stress. In addition, the Rubisco activation rate was decreased by ZnO treatment, while increased by melatonin treatment.

### 2.4. Effect of Melatonin on ATPase Activities under Nano-ZnO Stress

**Figure 6:**
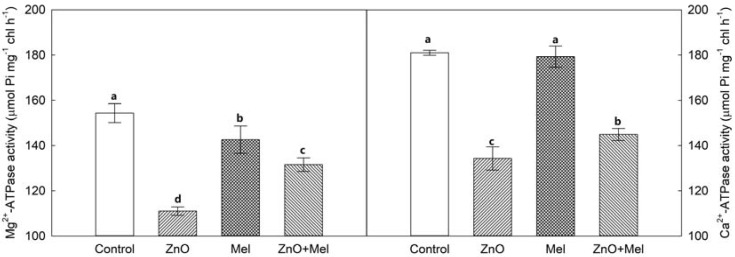
Activities of Mg^2+^-ATPase and Ca^2+^-ATPase in chloroplasts in the last fully expanded leaf in wheat as affected by melatonin application and Zinc Oxide (ZnO) nanoparticles. Mel, melatonin. Data represent the mean ± SE of three replicates. Different letters indicate significant differences (*p* < 0.05).

The activities of Mg^2+^-ATPase and Ca^2+^-ATPase were decreased by nano-ZnO treatment, compared with the control (Figure 6). Under the nano-ZnO stress, melatonin had a significant positive effect on the activities of Mg^2+^-ATPase and Ca^2+^-ATPase. However, melatonin had a significant negative effect on the activities of Mg^2+^-ATPase in wheat plants under non-stress condition.

### 2.5. Effect of Melatonin on H_2_O_2_ Concentration and Antioxidant Enzyme Activities under Nano-ZnO Stress

**Figure 7:**
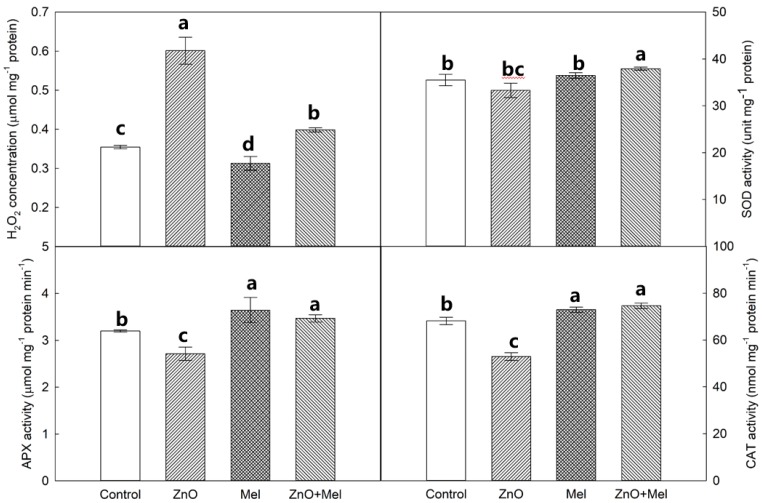
H_2_O_2_ concentration and activities of superoxide dismutase (SOD), ascorbate peroxidase (APX) and catalase (CAT) in the last fully expanded leaf in wheat as affected by melatonin application and Zinc Oxide (ZnO) nanoparticles. Mel, melatonin. Data represent the mean ± SE of three replicates. Different letters indicate significant differences (*p* < 0.05).

The highest H_2_O_2_ concentration in leaf was found in ZnO treatment, followed by the ZnO + Mel and the control, and the lowest value was in Mel treatment (Figure 7). Under non-stress condition, the melatonin treatment did not affect the SOD activity. However, it increased the superoxide dismutase (SOD) activity in plants exposed to nano-ZnO stress. Compared with the control, ZnO treatment decreased the ascorbate peroxidase (APX) activity; whereas, melatonin treatment increased the activity of APX. A similar trend as APX activity was found in the catalase (CAT) activity in wheat leaves.

## 3. Discussion

The toxic effects of nano-ZnO stress on plant growth has been reported to be caused by the ZnO NPs induced oxidative stress [35]. The parameters, including retardation in growth potential, biomass accumulation, and leaf area could be used for assessing the phytotoxicity of NPs in plants [5]. In the present study, the plant biomass accumulation and leaf area in wheat was reduced significantly by the nano-ZnO stress. Also, the total root length was significantly decreased in wheat plants exposed to nano-ZnO stress. Consistent with this, ZnO NPs (300 mg L^−1^) significantly reduced the elongation of root in *Arabidopsis* [1]. A number of reports are available which indicate the promoting effects of melatonin on plant growth and root elongation under both normal and stress conditions [36,37,38]. Here, a positive effect of melatonin application on the dry weight of shoot and root, leaf area, and root length in nano-ZnO stressed plants was also observed. The enhanced plant growth and biomass accumulation induced by melatonin was most likely due to the promoted photosynthetic C assimilation in wheat plants under nano-ZnO stress.

The photosynthetic C assimilation is related to chlorophyll concentration, electron transport efficiency, and Rubisco activity, which are all sensitive to the phytotoxicity of nanoparticles [2,4,39]. The chlorophyll limitation remarkably affects the photosynthetic C assimilation in plants under environmental stress [23]. In the present study, the ZnO NPs significantly decreased the Chl concentration in wheat leaves. However, the melatonin treated plants had significantly higher total Chl and Chl a concentrations than the non-melatonin treated plants, suggesting that melatonin has protective effects against ZnO NPs induced chlorophyll degradation in wheat leaves. The nano-ZnO stress induced decrease in Pn was larger in non-melatonin treated plants than that in melatonin treated plants, which was not related to the changes of stomatal conductance exposed to the interaction of melatonin and ZnO NPs, since no difference in *g_s_* was found between ZnO and ZnO + Mel treatments. Interestingly, ZnO NPs induced depression of photosynthesis could be, at least partly, attributed to the stomatal limitation. Thus, the alleviation effects of melatonin on photoinhibition under nano-ZnO stress were mainly attributed to the photosynthetic electron transport. The photosynthetic electron transport system is very sensitive to the phytotoxicity of nanoparticles, such as nano-CuO [2]. Here, the Fv/Fm and PI_ABS_ were both increased by melatonin in nano-ZnO stressed plants, indicating that melatonin has the positive effect on quantum yield of PSII and light absorption efficiency [40].

The leaf model of phenomenological energy fluxes per cross-section was used to visualize the derived parameters from the chlorophyll a fluorescence induction curve [14]. In the present study, the trapped energy flux per CS (TRo/CSm) and the electron transport in PS II cross-section (ETo/CSm) were significantly decreased in nano-ZnO stressed plants, indicated by smaller size of light blue and dark blue arrows in ZnO, compared with the control. However, melatonin treated plants had higher ETo/CSm than the non-melatonin treated plants under nano-ZnO stress. This indicated that the reoxidation of reduced Q_A_ through electron transport over a cross-section of active and inactive reaction centres (RCs) was improved by the melatonin application in wheat under nano-ZnO stress [41]. The density of the active RCs in PSII cross-section, which was indicated by open circles in Figure 4, was higher in ZnO + Mel treatment than that in ZnO treatment, implying that melatonin allowed less active state RCs were converted to inactive state of RCs, leading to less accumulation of electron via the photochemical processes under nano-ZnO stress. However, there was no significant difference in non-photochemical quenching (DIo/CSm) between ZnO and ZnO + Mel, which was indicated by the size of red arrows, showing that melatonin treatment did not affect the non-photochemical quenching in nano-ZnO stressed plants.

In C3 plants like wheat, Rubisco initiates the carbon assimilation by RuBP carboxylation [42]. The nano-ZnO stress decreased significantly the initial and total Rubisco activities and lowered the Rubisco activation. Notably, melatonin treatment reduced the Rubisco activities in wheat leaves under normal condition, but it enhanced the Rubisco activities under nano-ZnO stress. Similar results were found in barley under cold stress in our previous study [23]. Photosynthetic electron transport generates ATP and NADPH to drive the carbon reduction and photorespiratory C oxidation in the dark reaction in photosynthesis. In chloroplast, Mg^2+^-ATPase and Ca^2+^-ATPase play key roles in ATP formation [43]. In this study, the activities of these two functional enzymes were enhanced by melatonin in wheat exposed to nano-ZnO stress. Together with melatonin’s positive effects on electron transport and Rubisco activity, exogenous melatonin application could alleviate the nano-ZnO induced limitation in photosynthetic C assimilation in wheat.

It should be noted that ZnO NPs significantly increased the H_2_O_2_ concentration, while it decreased the activities of antioxidant enzymes in wheat leaves. Melatonin has been well reported as an antioxidant in plants, which could not only scavenge H_2_O_2_ directly but also regulate the antioxidant enzyme activities [44]. In the present study, melatonin treatment significantly enhanced the activities of antioxidant enzymes, including SOD, APX, and CAT, in the nano-ZnO stressed wheat plants. SOD catalyzes the disproportionation of single oxygen to produce H_2_O_2_ [45]. The H_2_O_2_ is then decomposed into H_2_O and O_2_ by the combination of CAT and APX [45]. The higher activities of these enzymes conferred the enhanced ROS scavenging capacity of wheat leaves exposed to nano-ZnO stress. This could also protect photosynthetic electron transport against the oxidative stress induced by nano-ZnO.

In conclusion, this study demonstrated that melatonin improved the photosynthetic C assimilation in nano-ZnO stressed wheat plants. This was mainly attributed to the higher chlorophyll concentration, enhanced activities of Rubisco and ATPases. Melatonin application also protected the photosynthetic electron transport system against the oxidative burst caused by nano-ZnO stress in wheat.

## 4. Materials and Methods

### 4.1. Experimental Setup

Winter wheat cv. Lianmai 6 were grown in plastic pots (15 cm in height and 25 cm in diameter). The pots were filled with 5 kg of clay soil moistened thoroughly with suspensions with 0 or 250 mg L^−1^ ZnO NPs (Dekedao nano Inc., Beijing, China) (particle size < 50 nm). Eight seeds were sown in each pot, and four seedlings remained after thinning at the third-leaf stage. At the fourth-leaf stage, half of the plants in each treatment were sprayed with 1 mM melatonin (50 mL per plant) for three days. Thus, four treatments were established: Control, non-ZnO stress; ZnO, ZnO stress treatment (250 mg L^−1^ ZnO NPs); ZnO + Mel, ZnO stress treatment + foliar melatonin treatment; Mel, foliar melatonin treatment. The measurements were conducted, and the leaf samples were collected after melatonin treatment. The plants were grown in a climate-controlled greenhouse at 26/16 °C (day/night). The photosynthetic active radiation (PAR, 12 h photoperiod and >500 μmol m^−2^ s^−1^). The experiment was a randomized block design, with three replicates for each treatment. Each replication consisted of six pots.

### 4.2. Total Root Length and Biomass

Total root length was analyzed with a WinRHIZO root analyser system (WinRHIZO 2012a, Regent Instruments Canada Inc., Montreal, QC, Canada). Shoot samples were oven-dried at 80 °C for 72 h and weighted to get dry mass.

### 4.3. Chlorophyll Concentration

Fresh leaf (0.1 g) was sliced and incubated in 50 mL of pigment extraction solution containing acetone and anhydrous ethanol (1:1, *v/v*) in the dark at 25 °C for 12 h. The supernatant was collected and measured for absorbance at 663 and 647 nm. Concentrations of total chlorophyll and chlorophyll a (Chl a) were calculated [46].

### 4.4. Gas Exchange, Chl a Fluorescence Transient, and Leaf Area

After the melatonin treatment, photosynthetic rate (Pn) and stomatal conductance (*g_s_*) of the last fully expanded leaves were taken for gas exchange measurement with a portable photosynthesis system (LI-6400, LI-Cor, Lincoln, NE, USA) at a CO_2_ concentration of 400 μmol mol^−1^ and photosynthetically active radiation of 1200 μmol m^−2^ s^−1^. Fast chlorophyll a fluorescence induction curve (JIP curve) was measured on the same leaf as for the gas exchange measurement using Plant Efficiency Analyzer (Pocket-PEA, Hansatech, Norfolk, UK). Before measuring, 30 min of dark adaptation of the leaf was applied. The data were processed and calculated using PEA Plus 1.04. Total leaf area was measured with a leaf area meter (LI-3100, Li-Cor Inc., Lincoln, NE, USA).

### 4.5. Rubisco Activity

Leaf samples (0.2 g) were ground in 40 mL of extraction buffer (50 mM Tris-HCl, 1 mM EDTA, 1 mM MgCl_2_, 10% PVP and 10 mM β-Mercaptoethanol), and then centrifuged at 15,000× *g* for 15 min. The supernatant was gently collected to measure Rubisco activities. Activities of Rubisco before (initial activity) and after (total activity) active site carbamylation were assayed by a spectrophotometric procedure coupled to NADH oxidation [23,47]. Rubisco activation was estimated as the percent ratio of initial to total activities for each sample.

### 4.6. Chloroplasts Isolation and ATPase Activity

Leaf samples (6 g) were ground in 30 mL of extraction buffer (0.45 M sucrose, 15 mM 3-(*N*-morpholino) propanesulfonic acid (MOPS), 1.5 mM ethylene glycol tetra acetic acid (EGTA), 0.6% polyvinylpyrro-lidone (PVP), 0.2% bovine serum albumine (BSA), 0.2 mM phenylmethylsulphonyl fluoride (PMSF) and 10 mM dithiothreitol (DTT)). Homogenate was filtered through eight layers of gauze and then the filtrate was centrifuged at 2000× *g* for 5 min. The sedimentation was used for chloroplast isolation. The sedimentation was resuspended with sorbitol resuspension medium (SRM, 0.33 M sorbitol in 50 mM 4-(2-hydroxyethyl)-1-piperazineethanesulfonic acid (HEPES)), and then layered on the top of a 7 mL layered system (35%, 80% Percoll) for the step gradients. The chloroplasts was collected and washed with 2 mL SRM followed by centrifugation at 1100× *g* for 10 min. Finally, the intact chloroplasts were kept with 2 mL SRM at −4 °C. The activities of Ca^2+^- and Mg^2+^-ATPase in the chloroplasts suspension were measured following the method of Li et al. [48].

### 4.7. H_2_O_2_ Concentration and Antioxidant Enzyme Activity

H_2_O_2_ concentration was measured by monitoring the absorbance of titanium peroxide complex at 410 nm following the methods of Zheng et al. [49]. APX (ascorbate peroxidase) activity was assayed following ascorbate oxidation by monitoring the decrease at 290 nm, the activity of SOD (superoxide dismutase) was measured by monitoring the inhibition of photochemical reduction of nitroblue tetrazolium (NBT) for 5 min. CAT (catalase) activity was measured as described by Li et al. [50].

### 4.8. Statistical Analysis

All data were subjected to the one-way analysis of variance (ANOVA) to determine the significant differences between treatments using the software of SigmaSATA (V3.5, Systat Software, San Jose, CA, USA).
